# Elevated Hemostasis Markers after Pneumonia Increases One-Year Risk of All-Cause and Cardiovascular Deaths

**DOI:** 10.1371/journal.pone.0022847

**Published:** 2011-08-10

**Authors:** Sachin Yende, Gina D'Angelo, Florian Mayr, John A. Kellum, Lisa Weissfeld, A. Murat Kaynar, Tammy Young, Kaikobad Irani, Derek C. Angus, for the GenIMS Investigators

**Affiliations:** 1 The Clinical Research, Investigation, and Systems Modeling of Acute Illness Center, University of Pittsburgh, Pittsburgh, Pennsylvania, United States of America; 2 Department of Critical Care Medicine, University of Pittsburgh, Pittsburgh, Pennsylvania, United States of America; 3 Division of Biostatistics, Washington University School of Medicine, St. Louis, Missouri, United States of America; 4 Division of Biostatistics, University of Pittsburgh, Pittsburgh, Pennsylvania, United States of America; 5 Division of Cardiology, University of Pittsburgh, Pittsburgh, Pennsylvania, United States of America; Fundação Oswaldo Cruz, Brazil

## Abstract

**Background:**

Acceleration of chronic diseases, particularly cardiovascular disease, may increase long-term mortality after community-acquired pneumonia (CAP), but underlying mechanisms are unknown. Persistence of the prothrombotic state that occurs during an acute infection may increase risk of subsequent atherothrombosis in patients with pre-existing cardiovascular disease and increase subsequent risk of death. We hypothesized that circulating hemostasis markers activated during CAP persist at hospital discharge, when patients appear to have recovered clinically, and are associated with higher mortality, particularly due to cardiovascular causes.

**Methods:**

In a cohort of survivors of CAP hospitalization from 28 US sites, we measured D-Dimer, thrombin-antithrombin complexes [TAT], Factor IX, antithrombin, and plasminogen activator inhibitor-1 at hospital discharge, and determined 1-year all-cause and cardiovascular mortality.

**Results:**

Of 893 subjects, most did not have severe pneumonia (70.6% never developed severe sepsis) and only 13.4% required intensive care unit admission. At discharge, 88.4% of subjects had normal vital signs and appeared to have clinically recovered. D-dimer and TAT levels were elevated at discharge in 78.8% and 30.1% of all subjects, and in 51.3% and 25.3% of those without severe sepsis. Higher D-dimer and TAT levels were associated with higher risk of all-cause mortality (range of hazard ratios were 1.66-1.17, p = 0.0001 and 1.46-1.04, p = 0.001 after adjusting for demographics and comorbid illnesses) and cardiovascular mortality (p = 0.009 and 0.003 in competing risk analyses).

**Conclusions:**

Elevations of TAT and D-dimer levels are common at hospital discharge in patients who appeared to have recovered clinically from pneumonia and are associated with higher risk of subsequent deaths, particularly due to cardiovascular disease.

## Introduction

It is well recognized that hospitalizations for community-acquired pneumonia (CAP) are associated with high long-term mortality, but underlying reasons are not known [1]–[6]. We and others have shown that higher long-term mortality for CAP cannot be solely explained by higher burden of chronic diseases prior to the occurrence of infection [6]–[8]. Rather, CAP may accelerate progression of pre-existing chronic diseases. For example, several epidemiologic studies have shown that acute cardiovascular events, including myocardial infarction [9]–[12], stroke [13], and pulmonary thromboembolism [14], occur at a higher than expected frequency following respiratory infections and are the most common cause of death after CAP [3], [6].

We recently proposed an alternative mechanism to explain the epidemiologic link between infection and the higher risk of death, particularly due to acute cardiovascular events [15]. Activation of the host response to infection may persist at hospital discharge when patients appeared to have recovered clinically from the infection, and increase risk of acute deterioration of cardiovascular disease and subsequent deaths. During an acute infection, activation of the hemostatic system leads to a prothrombotic state [16], [17]. Recently we showed that these abnormalities are common during less severe infection [16], [17]. Atherothrombosis may occur due to a prothrombotic state and lead to an acute cardiovascular event and death [18]. Therefore, in a large, multicenter cohort of survivors of CAP hospitalization we determined whether hemostatic abnormalities activated during an acute infection persist during recovery and increase risk of subsequent all-cause and cardiovascular deaths. We also assessed whether the association between circulating hemostasis markers and death was independent of previously reported association between increased circulating inflammatory marker levels and higher mortality [15].

## Methods

### Ethics Statement

The Institutional Review Boards at the following hospitals approved the study: Pennsylvania: Allegheny General Hospital, Jefferson Hospital/SHHS, Mercy Hospital, St. Clair Memorial Hospital, St. Francis Medical Center, Sewickley Valley Hospital, University of Pittsburgh Medical Center (UPMC) Braddock, UPMC Horizon, UPMC Lee, UPMC McKeesport, UPMC Passavant, UPMC Presbyterian, UMPC Shadyside, UPMC Southside, UPMC St. Margaret, West Penn Hospital; Connecticut: Bridgeport Hospital, Hartford Hospital, Milford Hospital, New Britain General Hospital, Norwalk Hospital, Yale-New Haven Hospital; Tennessee: Methodist Health Care (single IRB approval for three Methodist University sites); Michigan: Henry Ford Health System, Detroit Receiving/Sinai-Grace, Wayne State. Written, informed consent was obtained from all participants or by proxy.

### Subjects and design

We conducted 1-year follow-up of all hospital survivors of the Genetic and Inflammatory Markers of Sepsis (GenIMS) study [19]. GenIMS is a large, multicenter observational cohort of subjects with CAP presenting to the Emergency Departments (EDs) of 28 teaching and non-teaching hospitals in the US. Eligible patients were those older than 18 years of age with a diagnosis of CAP based on clinical and radiological criteria, as described by Fine et al [20]. Exclusion criteria included transfer from another hospital, discharge from an acute care hospital within the previous ten days, diagnosis of pneumonia within the previous 30 days, chronic dependency on mechanical ventilation, cystic fibrosis, active pulmonary tuberculosis, admission for palliative care, prior enrollment in the study, incarceration, and pregnancy.

### Clinical and outcome variables

We ascertained comorbid conditions using the Charlson comorbidity index [21] and pre-existing cardiovascular disease by patient report and chart review. APACHE III and the Pneumonia Severity Index (PSI) were calculated to assess illness severity [20], [22]. We defined severe sepsis as pneumonia with acute organ dysfunction following the 2001 International Consensus Criteria [23], [24]. We assessed vital signs at hospital discharge to determine whether subjects met criteria for clinical stability at hospital discharge, as described by Halm et al [25].

The primary outcome variables were all-cause and cardiovascular (death due to acute myocardial infarction, ischemic or atherosclerotic heart disease, and cerebrovascular disease) mortality 1 year after hospital discharge. We ascertained all-cause and cause-specific mortality using the National Death Index (NDI) search and NDI-coded causes of death [26]. The sensitivity and specificity of NDI to assess deaths is >96% [27]. Causes of death assessed by NDI matched causes of death assessed by nosologists in >93% of cases in prior studies [28], [29].

### Laboratory procedures

We obtained blood daily for the first week and once weekly thereafter, while subjects remained in hospital. For this study we analyzed the last available hemostasis markers, including antithrombin (AT), Factor IX, thrombin-antithrombin (TAT) complexes, plasminogen activator inhibitor (PAI)-1, and D-dimer, and inflammatory marker (interleukin [IL]-6).

We analyzed coagulation and fibrinolysis markers by a commercial laboratory (Esoterix, Agoura Hills, CA, USA). Specific methods and kits used were: D-dimer, latex immunoassay (Diagnostica Stago, Parsippany, NJ, USA); PAI-1, bio immunoassay (Biopool Chromolize, Biopool International, Ventura, CA, USA); AT, chromogenic (BioMerieux, Rhône-Alpes, France); Factor IX, clot (BioMerieux); and TAT, ELISA (Behring, King of Prussia, PA, USA). Abnormal values were defined by the clinical laboratory or manufacturer's assay. These abnormalities included: D-dimer >256 ng/ml, PAI-1 activity >31 IU/ml, antithrombin activity <70%, Factor IX activity <60%, and TAT >5.0 ng/ml. We measured IL-6 concentrations by chemiluminescent immunoassay using an automated analyzer (IMMULITE, Diagnostic Products Corp., Los Angeles, CA, USA).

### Statistical analyses

We first conducted univariate comparisons for subjects who survived or died at 1 year. We assumed a log-normal distribution for all markers and analyzed data in natural log scale. We assessed the association between each marker and risk of death over 1 year using the Gray's survival model [30]. We used the Gray's survival models to estimate hazard ratios (HR) because the hazards failed Cox's proportionality assumption. The Gray's model estimates HR across 10 time intervals and provides a detailed description of the change in hazard ratios over 1 year. We determined unadjusted and adjusted HRs, adjusting for age, sex, race, and Charlson score.

We conducted several sensitivity analyses. We analyzed the subgroups that were less severely ill and most likely to have recovered from the infection by analyzing those without severe sepsis and those who were discharged home. Since pre-existing cardiovascular disease is a potential confounder, we repeated the analyses in the subset who did not report cardiovascular disease.

We assessed the association between each marker and cardiovascular deaths, using a competing risk model because the associations between these markers and different causes of death are dependent and these risks compete with one another until a subject dies due to a specific cause of death [31]. Previously, we showed that inflammatory markers that are activated during the acute infection remain elevated at hospital discharge [15], and higher levels of IL-6 at discharge were associated with higher risk of cardiovascular events. We therefore determined whether the association between hemostasis markers and 1-year mortality were independent of previously reported association between IL-6 and 1-year mortality.

Finally, we used logistic regression and calculated area under curve (AUC) to evaluate the discriminatory ability of biomarkers to predict long-term mortality and cardiovascular deaths. We limited these analyses to biomarkers that were significant in the multivariable analyses (IL-6 and D-dimer), and assessed AUC for biomarkers alone and for biomarkers and pre-infection clinical factors.

## Results

### Baseline characteristics and hospital course

**Figure 1:**
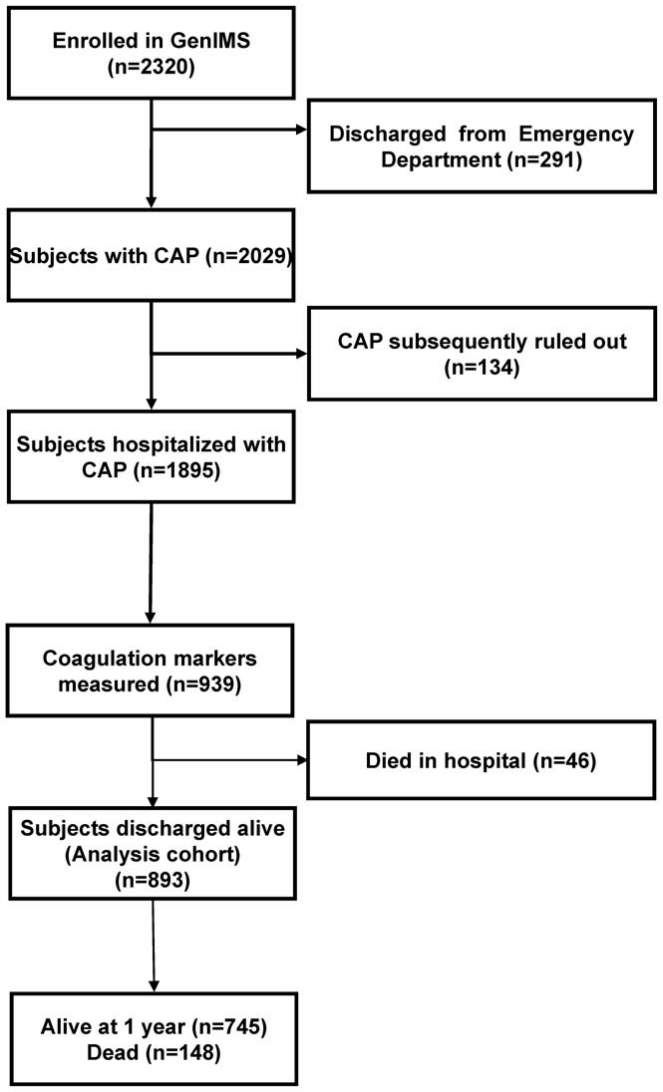
Subject disposition for the entire Genetic and Inflammatory Markers of Sepsis (GenIMS) cohort. CAP indicates community-acquired pneumonia.

Of the 2320 subjects enrolled in GenIMS, 291 patients were excluded because they were discharged from the ED and another 134 were excluded because the clinical team ruled out CAP during the first three days of hospitalization (Figure 1). Of the remaining 1895 subjects, hemostasis markers were measured in a random subset of 939 consecutive subjects (49.5%). Of these, 46 (4.9%) died in the hospital, and we conducted our analysis on 893 subjects who were discharged alive.

Pre-hospitalization clinical characteristics, severity of illness, and hospital course were similar for all subjects who were discharged alive (n = 1799) and the subset (n = 893) who had hemostasis markers measured at hospital discharge (Table 1). For the latter group, the mean age of all subjects was 68.7 years, approximately half were female, and two-thirds had at least 1 comorbid condition based on the Charlson score. Pre-existing cardiovascular disease was found in 201 (22.5%) subjects. Most subjects were non-Hispanic whites (86.6%), an additional 94 (10.5%) subjects were blacks, and the remaining 25 subjects represented other races. The majority of subjects never required admission to the intensive care unit (n = 773, 86.6%) and never developed severe sepsis (n = 631, 70.6%), and half (n = 417, 46.7%) of the subjects had a PSI score less than 90 (PSI class I–III), suggesting that most subjects did not have severe pneumonia. Mean hospital stay was 7 days. Three quarters of subjects (n = 672, 75%) were discharged home and the remaining were discharged to acute or sub-acute care facilities or residential facilities.

**Table 1:** Demographic and clinical characteristics.

	All subjects discharged alive (n = 1799)	Subset with hemostatic markers (n = 893)	Subset with hemostatic markers stratified by 1-year survival		
Variable			Non survivors (n = 148)	Survivors (n = 745)	P value
Demographics					
Age, mean (sd, median)	67 (17, 71)	68.7 (15, 73)	76.5 (11, 78)	67.1 (16, 71)	<0.0001
Sex, female, n (%)	867 (48.2)	436 (48.8)	57 (38.5)	379 (50.8)	0.006
Race, white, n (%)	1443 (80.2)	774 (86.6)	132 (89.1)	642 (86.1)	0.32
Charlson comorbidity score>0	1297 (72.1)	616 (68.9)	123 (83.1)	493 (66.1)	<0.0001
Cardiovascular disease, n (%)	464 (29.1)	201 (22.5)	45 (30.4)	156 (20.9)	0.01
Severity of illness					
Day 1 PSI*, mean (sd, median)	97 (36, 95)	95.5 (33, 93)	117.4 (32, 113)	91.1 (31, 90)	<0.0001
PSI Class I and II, n (%)	424 (23.6)	204 (22.9)	8 (5.4)	196 (26.3)	<0.0001
PSI Class III, n (%)	389 (21.6)	213 (23.9)	23 (15.5)	190 (25.5)	
PSI Class IV, n (%)	663 (36.9)	340 (38)	69 (46.6)	271 (36.3)	
PSI Class V, n (%)	323 (18.0)	136 (15.2)	48 (32.4)	88 (11.8)	
Day 1 APACHE III score, mean (sd, median)	55 (17, 54)	55.8 (16, 55)	64.8 (15, 64)	53.9 (15, 53)	<0.0001
Severe sepsis, n (%)	498 (27.7)	262 (29.3)	71 (47.9)	191 (25.6)	<0.0001
Hospital course					
Need for mechanical ventilation, n (%)	90 (5.0)	37 (4.1)	4 (2.7)	33 (4.4)	0.33
Required ICU stay, n (%)	250 (13.9)	120 (13.4)	19 (12.8)	101 (13.5)	0.81
Length of hospital stay, mean (sd, median)	7 (5,6)	7.2 (5, 6)	8.3 (5, 7)	7 (5, 6)	0.0001
Location following hospital discharge					
Home, n (%)	1374 (77.5)	672 (75.3)	84 (56.8)	588 (78.9)	<0.0001
Acute or sub-acute care facility, n (%)	159 (9.0)	101 (11.3)	24 (16.2)	77 (10.3)	
Residential care facilities, n (%)	43 (2.4)	101 (11.3)	36 (24.3)	65 (8.8)	
Others, n (%)	6 (0.4)	19 (2.1)	4 (2.7)	15 (2)	

*Based on Pneumonia severity index by Fine et al. [20].

Fifty-eight (6.4%), 94 (10.5%), and 148 (16.5%) subjects died by 90 days, six months, and 1 year, respectively. Of the 148 deaths over 1 year, 33 (22.9%) were due to cardiovascular causes. Most subjects who died due to cardiovascular causes did not report history of cardiovascular disease (n = 20, 60.6% did not report pre-existing cardiovascular disease).

Compared to 1-year survivors, non-survivors were older men with at least 1 comorbid condition and they were more likely to report cardiovascular disease prior to developing CAP (30.4% vs. 20.9%, P = 0.01, Table 1). Non-survivors had more severe CAP on presentation, as evidenced by higher PSI and APACHE III scores, were more likely to have developed severe sepsis, and incurred a longer duration of hospital stay.

### Hemostasis markers at hospital discharge

Most subjects met the Halm criteria for clinical stability on the day of discharge. Only 15 (1.6%), 73 (8.1%), 24 (2.6%), and 8 (0.9%) subjects had a temperature greater than 37.8 C, heart rate greater than 100/minute, respiratory rate greater than 24/minute, or systolic blood pressure <90 mm of Hg, respectively. All four vital signs were normal in 789 (88.4%) subjects, 1 vital sign was abnormal in 89 (9.9%) subjects, and only 15 (1.7%) subjects had abnormalities in two or more vital signs.

Hemostasis markers were measured on the day of discharge in 523 (58.5%) subjects and most subjects (n = 805, 90.5%) had hemostasis markers measured within 96 hours of discharge. For Factor IX, AT, TAT, PAI-1, and D-dimer, the average circulating concentrations (geometric means) at hospital discharge were 117.8%, 91.5%, 3.6 ng/ml, 3.8 IU/ml, and 598.3 ng/ml, respectively (Table 2). Circulating TAT and D-dimer concentrations were elevated in 30.1% and 78.8%, whereas levels had returned to normal for most subjects for Factor IX, AT, and PAI-1 (7.9%, 10%, and 5% had abnormal levels).

**Table 2:** Mean, median, interquartile range, and percentage of subjects with abnormal hemostasis marker levels at hospital discharge.

Hemostasis markers	Geometric mean	Median and interquartile range	Percentage with abnormal levels*		
			All subjects (n = 893)	Without severe sepsis (n = 631)	Discharged home (n = 672)
Factor IX (%)	117.8	129 (101, 158)	7.9	7.1	8
Antithrombin (%)	91.5	93 (80, 105)	10	8.8	8.3
TAT complexes (ng/ml)	3.6	3.3 (2.0, 5.9)	30.1	25.3	25.1
PAI-1 (IU/ml)	3.8	3.7 (1.0, 8.8)	5	5.3	5.2
D-dimer (ng/ml)	598.3	596 (298, 1210)	78.8	51.3	53.4

*Abnormal values were defined by the clinical laboratory or manufacturer's assay. These abnormalities included: D-dimer >256 ng/ml, PAI-1 activity >31 IU/ml, antithrombin activity <70%, Factor IX activity <60%, and TAT >5.0 ng/ml.

The high frequency of elevated TAT and D-dimer levels at hospital discharge were consistently observed across various subgroups without severe pneumonia (Table 2). For example, TAT and D-dimer levels were elevated in 25.3% and 51.3% of subjects without severe sepsis and 23.5% and 49.4% of subjects with less severe pneumonia (PSI class I–III). Furthermore, TAT and D-dimer levels were elevated in 25.1% and 53.4% of subjects who were discharged home from the hospital and likely to have recovered from the acute infection.

### Hemostasis markers and all-cause mortality

**Figure 2:**
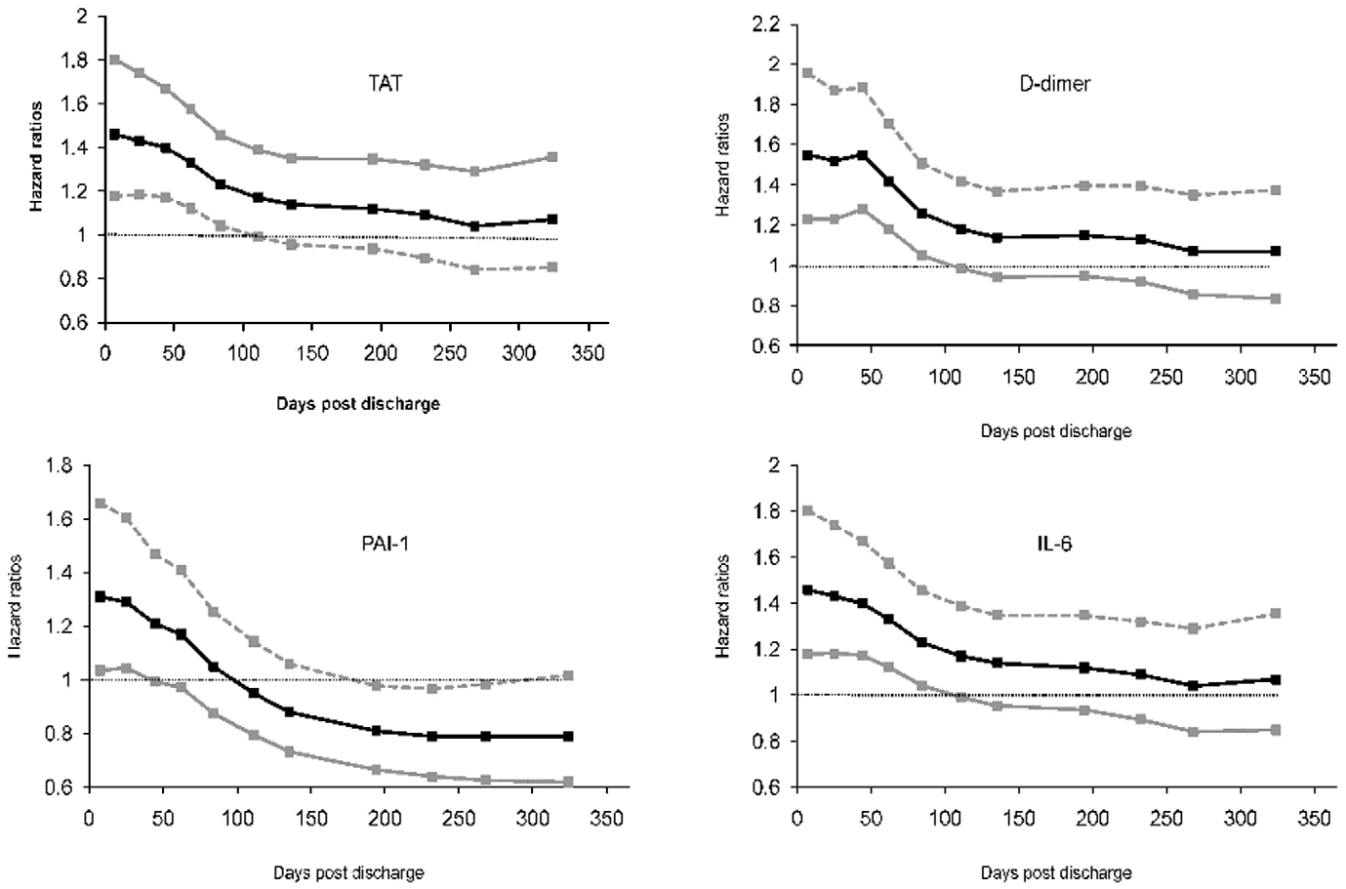
Varying hazard ratios with 95% confidence intervals (CI) for circulating thrombin-antithrombin (TAT) complexes, D-dimer, plasminogen activator inhibitor (PAI)-1, and interleukin (IL)-6 concentrations measured at hospital discharge and mortality over 1 year. The hazard ratios are shown over ten time intervals based on the Grays' model. For TAT, D-dimer, and IL-6, the lower bounds of the CI are above 1 for the first 111 days. For PAI-1, the lower bounds of the 95% CI are above 1 for the first 44 days.

**Figure 3:**
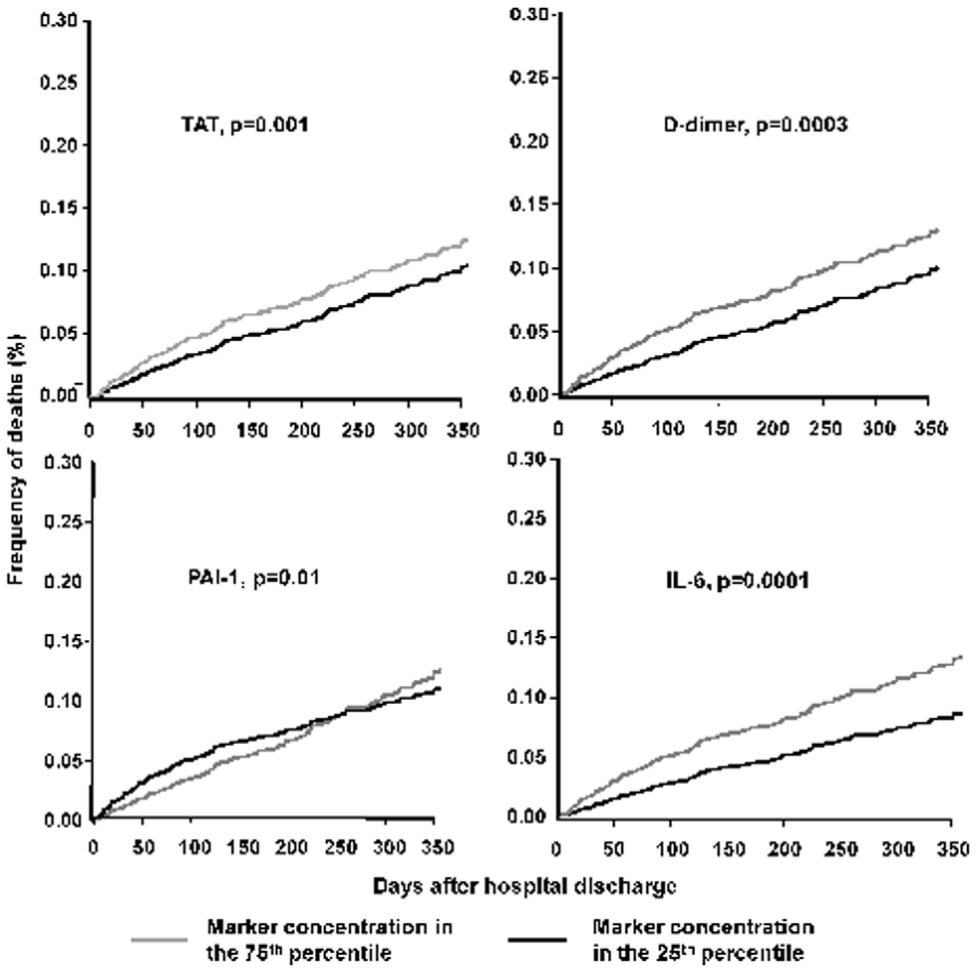
Failure plots for high and low concentrations (25^th^ and 75^th^ percentiles of marker concentrations) of thrombin-antithrombin (TAT) complexes, D-dimer, plasminogen activator inhibitor (PAI)-1, and interleukin (IL)-6 concentrations measured at hospital discharge and mortality over 1 year. Using the Gray's model, the hazard ratios are estimated at ten intervals over 1 year and hazard ratios over five representative periods are shown. P values are obtained from the Grays' survival model.

In univariate analyses, circulating concentrations of each hemostasis marker at hospital discharge was associated with 1-year survival. Table 3 and Figure 2 describes the range of HR over ten time intervals for each hemostasis marker and risk of death over 1 year. For example, the range of HRs for D-dimer was 1.6-1.1 (p = 0.0001). The hazard of death for subjects with high D-dimer levels were highest initially (HR = 1.6 during the initial week), decreased over time, and the HR was 1.1 during the last 3 months. A similar pattern was observed for TAT where higher circulating levels was associated with higher 1-yr risk of death (range = 1.49-1.1, p = 0.0001). The lower bounds of the 95% CI for the hazard ratios remained above 1 for the first 111 days for TAT and D-dimer. For PAI-1, the higher risk was observed only during the initial 44 days. When adjusted for age, sex, race, and Charlson score, higher circulating concentrations of TAT, PAI-1, and D-dimer were associated with higher 1-year mortality (Table 3). Figure 3 show failure plots for 25^th^ and 75^th^ percentile of circulating TAT, D-dimer, and PAI-1 and survival over 1 year.

**Table 3:** Hazard ratios* for association between circulating hemostasis markers at hospital discharge and all-cause mortality over 1 year.

Hemostasis markers	Unadjusted hazard ratios	P value	Adjusted hazard ratios†	P value
Factor IX	0.56–0.74	0.005	0.68–0.94	0.23
Antithrombin	0.2–0.65	0.029	0.38–1.43	0.59
TAT complexes	1.1–1.49	0.0001	1.04–1.46	0.001
PAI-1	0.73–1.17	0.008	0.79–1.31	0.011
D-dimer	1.1–1.6	0.0001	1.07–1.55	0.0003
IL-6	1.21–1.68	<0.0001	1.17–1.66	0.0001

*Range of hazards ratios are reported for ten time intervals over 1 year using the Gray's model [30];

† Adjusted for age, race, sex, and Charlson score.

Consistent with prior results [15], higher concentrations of IL-6 were associated with higher risk of 1-year mortality (Table 3). Similar to the hemostasis markers, the hazard ratios were highest at hospital discharge and decreased over time (adjusted range of hazard ratios were 1.66-1.17, p = 0.0001, Figure 2 and 3). IL-6 concentrations significantly correlated with TAT and D-dimer levels, but the magnitude of the correlation coefficients were modest (Pearson coefficients for TAT and D-dimer with IL-6 were 0.16 and 0.32, p<0.0001 for both). The association between higher TAT and D-dimer levels and 1-year mortality were unchanged when adjusted for IL-6 levels at hospital discharge (range of HR = 1.4-1.01, p = 0.009 and 1.46-1.0, p = 0.008 for TAT and D-dimer).

The presence of severe sepsis during hospitalization did not influence the associations between each hemostasis markers and 1-year survival (interaction p value>0.05 for all markers, Table 4). The hazard ratios for PAI-1, TAT, and D-dimer were similar in subjects with and without severe sepsis but the confidence intervals were wider. We also performed sensitivity analyses by restricting the analyses to subjects with a hemostasis marker measurement within 48 hours (n = 735), those discharged home (n = 672), and those who did not report pre-existing cardiovascular disease (n = 692). In these analyses, the hazard ratios remained unchanged for the associations between TAT, D-dimer, PAI-1, and IL-6 and reduced 1-year survival (Table 4), though the association with mortality did not meet statistical significance for some comparisons.

**Table 4:** Sensitivity analyses for association between circulating hemostasis marker concentrations at hospital discharge and mortality over 1 year.

Hemostasis markers	Adjusted hazard ratios†	P value
Subjects in whom hemostasis markers were measured within 48 hours (n = 735)		
TAT complexes	1.04–1.41	0.022
PAI-1	0.9–1.42	0.091
D-dimer	1.07–1.64	0.0008
IL-6	1.26–1.81	<0.0001
Subjects who did not report cardiovascular disease (n = 692)		
TAT complexes	1.03–1.41	0.032
PAI-1	0.76–1.36	0.022
D-dimer	1.08–1.45	0.017
IL-6	1.24–1.74	0.0001
Subjects without severe sepsis (n = 631)		
TAT complexes	0.94–1.3	0.39
PAI-1	0.86–1.37	0.14
D-dimer	0.97–1.55	0.023
IL-6	1.26–2.18	<0.0001
Subjects with severe sepsis (n = 262)		
TAT complexes	1.1–1.62	0.014
PAI-1	0.65–1.26	0.069
D-dimer	1.0–1.55	0.11
IL-6	0.9–1.12	0.86
Subjects discharged home (n = 672)		
TAT complexes	1.02–1.21	0.59
PAI-1	0.77–1.33	0.12
D-dimer	0.89–1.43	0.18
IL-6	1.17–1.68	0.0017

*Range of hazards ratios are reported for ten intervals over 1 year using the Gray's model [30];

† Adjusted for age, race, sex, and Charlson score.

### Hemostasis markers and cause-specific mortality

Cardiovascular disease and cancer were the most common causes of death over 1 year and each accounted for approximately a fourth of all deaths (Table 5). Infections, renal failure, and chronic respiratory disease accounted for 10.4% (n = 15), 7.6% (n = 11), and 14.5% (n = 21) of deaths, respectively. Table 5 shows the geometric means for TAT, D-dimer, and PAI-1 for each cause of death. Among subjects who died subsequently due to cardiovascular disease, the D-dimer and TAT levels were highest (1100.9 ng/ml and 5.5 ng/ml) and most had D-dimer levels that were two-fold higher than levels seen in healthy individuals (87.8% and 81.8% had D-dimer levels >256 ng/ml and >512 ng/ml). In competing risk analyses, higher circulating TAT and D-dimer concentrations at hospital discharge were associated with deaths due to cardiovascular diseases (P = 0.003 and 0.009) but PAI-1 levels were not associated with cardiovascular deaths (P = 0.30).

**Table 5:** Association between hemostasis markers at hospital discharge and cause-specific mortality over 1 year*.

Causes of death over 1 year	%, (n)	Geometric means of inflammatory markers at hospital discharge			
		TAT	PAI-1	D-Dimer	IL-6
Cardiovascular†	22.3 (33)	5.5	3.5	1100.9	8.8
Infection‡	10.4 (15)	4.3	2.4	644.9	12.0
Cancer	29.8 (43)	4.2	2.8	912.5	14.5
Chronic lower respiratory disease	14.5 (21)	5.4	2.7	573.7	6.1
Renal failure	7.6 (11)	5.1	3.3	746.5	13.0
Others	9.7 (14)	4.5	4.2	880.3	8.3

† Cardiovascular causes of death include death due to acute myocardial infarction, ischemic or atherosclerotic heart disease, and cerebrovascular disease.

### Biomarkers and predicting long-term mortality

Table 6 shows AUC for IL-6 and D-dimer to predict l-year all-cause and cardiovascular mortality with and without clinical factors. The discriminative ability of IL-6 and D-dimer was modest (AUC = 0.60 and 0.58 for 1-year mortality for IL-6 and D-dimer) and AUC improved after adding clinical risk factors, including age, sex, race, and Charlson comorbidity score (AUC = 0.72 for both IL-6 and D-dimer). Similar results were observed for 1-year cardiovascular mortality, though D-dimer had better discriminative ability (AUC = 0.57 and 0.62 for IL-6 and D-dimer).

**Table 6:** Area under curve (AUC) to determine discriminative ability of interleukin (IL)-6 and D-dimer to predict long-term outcomes.

Biomarker	1-year all-cause mortality		1-year cardiovascular mortality	
	Without clinical factors	With clinical factors*	Without clinical factors	With clinical factors*
IL-6	0.60	0.72	0.57	0.74
D-dimer	0.58	0.72	0.62	0.76

*Clinical factors were age, sex, race, and Charlson comorbidity score.

## Discussion

In a large cohort of patients hospitalized with pneumonia, we have shown that hemostasis markers are elevated during recovery, as evidenced by higher TAT and D-dimer levels at hospital discharge. At least a third and half of the cohort had higher TAT and D-dimer levels at hospital discharge, even when analyses were restricted to subjects who experienced less severe illness during the hospital course or to those that appeared to have recovered clinically and were discharged home from the hospital. Higher concentrations of these hemostasis markers were associated with higher risk of death over 1 year, particularly due to acute deterioration of cardiovascular disease. These results were independent of previously reported associations between inflammatory markers and cardiovascular deaths [15]. Our results suggest that a persistent prothrombotic state after infection may explain the epidemiologic link between infection and higher risk of cardiovascular disease. Thus, interventions, such as aspirin and statins, with beneficial effects on resolution of the prothrombotic state and inflammation, should arguably be investigated to improve long-term outcomes after pneumonia [32], [33].

Our results are consistent with a previous animal study showing that hemostasis markers are elevated after infection. In a mouse model of influenza, higher circulating concentrations of TAT complexes, D-dimer, and PAI-1 persisted up to two weeks after the infection [34]. Our results are also consistent with small preliminary clinical studies [35]–[37]. For instance, higher hemostatic marker concentrations have been reported during winter months when the incidence of respiratory infections is high [35]–[37]. Longitudinal analysis of hemostatic markers has been performed in a small study of 54 subjects, where these markers were measured prior to, during, and two weeks after a respiratory tract infection and showed higher levels during recovery, but results were not statistically significant [36].

Most individuals who died due to cardiovascular events in our study did not have a prior history of cardiovascular disease. We speculate that many subjects had sub-clinical cardiovascular disease. Persistent inflammation and a pro-thrombotic state after pneumonia may lead to activation of atherosclerotic plaque, plaque rupture, and atherothrombosis, thereby precipitating an acute cardiovascular event.

Higher levels of biomarkers on average were associated with worse outcomes in our study, but biomarkers did not improve discriminative ability to predict long-term outcomes. The lack of improvement in discriminative ability could be due to the spread of the biomarker values. The AUC for models incorporating biomarkers and clinical risk factors was higher compared to models that incorporated biomarkers alone, suggesting that clinical factors may be important predictors. Additional studies using different approaches, such as net reclassification index [38], will be needed to understand the incremental value of measuring biomarkers for different clinical risk groups. Our observations provide important insights into a potential biologic link between acute inflammation and subsequent death, but biomarkers may not be useful clinical diagnostic tools.

In sensitivity analyses, we observed that hazard ratios for the association between IL-6 and 1-year mortality were larger in the subgroup without severe sepsis than among those with severe sepsis. No interaction was observed between IL-6, severe sepsis occurrence, and 1-year mortality. The clinical significance for these intriguing findings is unclear. A potential explanation could be that patients who have higher IL-6 concentrations but did not meet clinical criteria for severe sepsis had more severe disease and therefore associated with higher mortality.

In addition to deaths due to cardiovascular causes, deaths due to cancer accounted for approximately a third of all deaths. Deaths due to cancer may be increased in survivors of an acute infection due to multiple reasons, including identification of co-existing cancers during radiologic investigations to diagnose pneumonia, inability to tolerate chemotherapeutic regimens due to deconditioning during recovery, and progression of cancer due to increased systemic inflammation. The latter may occur due to genetic and epigenetic modifications of cells [39].

Our study has several strengths. First, we analyzed a large cohort of subjects who were recruited from different sites. We analyzed patients hospitalized for pneumonia because prior studies have shown an association between respiratory tract infection and increased risk of cardiovascular events [9]–[11]. Thus, our results are less likely to be confounded by differences in nature of infection. Second, higher TAT and D-dimer levels and their association with mortality and cardiovascular disease were robust across different subgroups, including those with less severe illness and subjects who had recovered adequately to be discharged home from the hospital. Most subjects had normal vital signs at hospital discharge. Thus, our results are less likely to be confounded by illness severity or due to ongoing organ dysfunction at hospital discharge.

Our study has limitations. First, we assessed only fatal cardiovascular events. Whether elevated hemostasis markers are associated with non-fatal cardiovascular events after hospital discharge is not known. Second, for practical reasons, we analyzed a small number of hemostasis markers. We chose these markers because they are activated during infection and were associated with higher risk of cardiovascular events in prior studies [35], [40]. Since subjects were recruited from several sites, we were unable to measure platelet function to accurately assess the prothrombotic state because these assays have to be performed promptly after collecting a blood sample. Furthermore, other markers within the coagulation pathway and markers of endothelial function may also play an important role. Third, pre-infection levels of D-dimer were not available in our study. However, in most subjects the D-dimer levels were at least two-fold higher than the manufacturer's upper limit of normal and were four-fold higher among those who died due to cardiovascular causes. Such high levels have been reported in patients with suspected pulmonary thromboembolism and deep venous thrombosis [41]. Thus, our results are unlikely to be confounded by higher pre-infection levels.

In conclusion, our results suggest a mechanism to explain increased long-term mortality after pneumonia hospitalization and the well-described epidemiologic link between infection and cardiovascular disease. A prothrombotic state is common at hospital discharge in patients recovering after pneumonia hospitalization and is associated with higher risk of all-cause mortality and cardiovascular deaths over 1 year after hospital discharge.
